# Reducing Emotional Distress for Childhood Hypoglycemia in Parents (REDCHiP): Protocol for a Randomized Clinical Trial to Test a Video-Based Telehealth Intervention

**DOI:** 10.2196/17877

**Published:** 2020-08-18

**Authors:** Susana R Patton, Andrew McConville, Arwen M Marker, Alexandra D Monzon, Kimberly A Driscoll, Mark A Clements

**Affiliations:** 1 Nemours Children’s Health System Jacksonville, FL United States; 2 Clinical Child Psychology Program University of Kansas Lawrence, KS United States; 3 Department of Clinical and Health Psychology University of Florida Gainesville, FL United States; 4 Children's Mercy Hospital–Kansas City Kansas City, MO United States

**Keywords:** diabetes mellitus, type 1, telemedicine, eHealth, child, parents, hypoglycemia, fear

## Abstract

**Background:**

Despite the introduction of new insulin analogs, insulin pumps, and continuous glucose monitoring (CGM), young children with type 1 diabetes mellitus (T1D) remain vulnerable to episodes of hypoglycemia because of their unpredictable eating and activity patterns and high degree of insulin sensitivity. Caregivers and young children living with T1D learn to fear hypoglycemia because it is uncomfortable, unpredictable, and dangerous. Up to 60% of caregivers of young children with T1D report moderate to severe levels of fear of hypoglycemia, and caregiver fear of hypoglycemia relates to lower quality of life for families and suboptimal child glycemic control. Yet, until recently, there have been no studies reporting on a targeted intervention to treat caregiver fear of hypoglycemia in families of young children.

**Objective:**

The aim of this project is to conduct a randomized clinical trial of an innovative, video-based telehealth intervention to treat fear of hypoglycemia in caregivers of young children with T1D versus a relevant, age-appropriate attention control intervention.

**Methods:**

We created the Reducing Emotional Distress for Childhood Hypoglycemia in Parents (REDCHiP) intervention by merging age-appropriate T1D education and behavioral parenting strategies with cognitive behavioral therapy strategies that are effective for reducing fear and promoting adaptive coping. REDCHiP uses 10 video-based telehealth sessions that are a combination of group and individual sessions. We will recruit up to 180 families of young children with T1D to participate in this clinical trial from two pediatric diabetes clinics located in the midwestern and southern United States. Once families have been enrolled, we will randomize caregivers based on child age (age 2-3 years or 4-5 years), child sex, and family CGM use to participate in the REDCHiP or attention control intervention. Families will complete 3 assessment visits that coincide with study entry, end of treatment, and 3-month posttreatment. At each assessment visit, we will collect questionnaire data from caregivers, accelerometry data from caregivers and children, CGM data from children, and a blood sample to measure glycated hemoglobin levels from children.

**Results:**

Recruitment began in July 2019, and enrollment is ongoing. The first wave of intervention delivery began in December 2019. We anticipate completing enrollment in 2023. Final reporting of results will occur within 12 months of the primary completion date.

**Conclusions:**

If the REDCHiP intervention is efficacious, next steps will be to examine multiple implementation strategies to determine how best to disseminate the intervention to pediatric diabetes clinics around the world.

**Trial Registration:**

ClinicalTrials.gov NCT03914547; https://clinicaltrials.gov/ct2/show/NCT03914547

**International Registered Report Identifier (IRRID):**

PRR1-10.2196/17877

## Introduction

### Background

Type 1 diabetes mellitus (T1D) is a common pediatric chronic condition characterized by the loss of natural insulin production and inability to regulate blood glucose levels. Incidence rates of T1D are increasing in children aged younger than 6 years internationally [1], and data also demonstrate a 1.7% average annual increase of new cases among children aged 5 to 9 years in the United States specifically [2]. Achieving near-normal glycemic control (glycated hemoglobin [HbA_1c_] <7.5%) early in the course of T1D may reduce economic burden to the health care system, and most importantly, can reduce the risk for later T1D-related complications [3]. This goal, however, is difficult for young children with T1D and their families to achieve as only 20% of young children in the United States and 58% of young children in Europe currently meet the HbA_1c_ target of <7.5% [4]. Managing T1D is particularly challenging in young children because of their heightened insulin sensitivity, unpredictable eating behaviors, inconsistent physical activity levels, and limited communication skills [5], which lead to extreme glycemic variations and severe hypoglycemic episodes [6].

Hypoglycemia events, or low blood glucose levels, are an immediate and dangerous complication of T1D [7]. Symptoms of hypoglycemia may include headaches, dizziness, impaired consciousness, irritability, weakness, sweating, racing pulse, and, in extreme cases, seizure, coma, or death [8,9]. Prevalence rates for severe hypoglycemia in young children with T1D are 2-fold higher than older children and adolescents and 3-fold higher than adults [10]. Not surprisingly, it is common for caregivers of young children with T1D to report elevated stress and anxiety regarding the probability of their child experiencing a hypoglycemia event [11]. Unfortunately, the introduction of new technologies such as shorter and longer acting insulin analogs, insulin pumps, and continuous glucose monitors (CGMs) have not eliminated the occurrence of hypoglycemia events in young children [11-13] or reduced caregiver fear of hypoglycemia [11,14].

**Figure 1:**
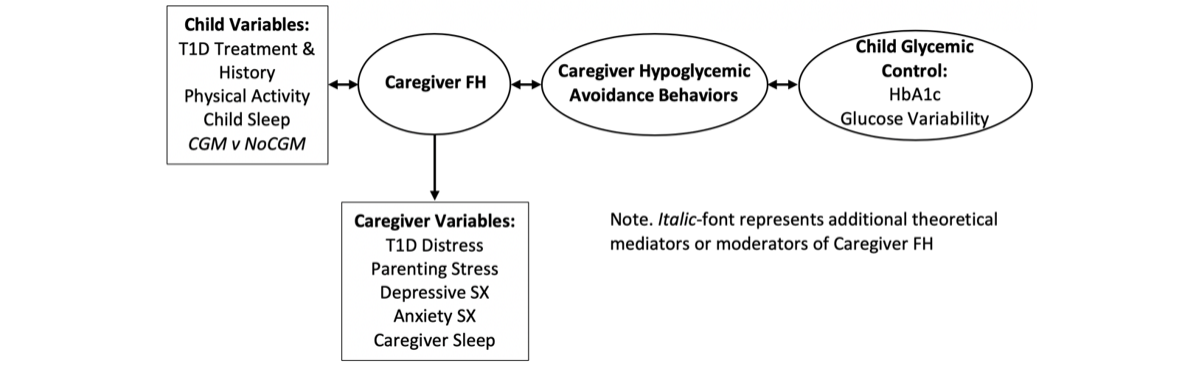
Theoretical model of caregiver fear of hypoglycemia.

Up to 60% of caregivers of young children with T1D report fear of hypoglycemia, which directly affects caregiver psychological and emotional well-being [15]. Additionally, in young children with T1D, caregivers may perceive hypoglycemia as unpredictable, exacerbating their level of fear [16]. Building on past research, we developed a theoretical model for caregiver fear of hypoglycemia (Figure 1) which identifies child and caregiver variables that may underlie caregiver fear of hypoglycemia. For example, we have data suggesting that a child’s T1D history, including past experience with hypoglycemia, and a child’s sleeping behavior may relate to caregiver fear of hypoglycemia [15,17,18]. In addition, in older youth with T1D, there is evidence that child physical activity levels relate to caregiver fear of hypoglycemia [19-22].

For caregiver variables, several studies suggest that T1D-related distress, parenting stress, caregiver depressive and anxiety symptoms, and decreased caregiver sleep may exacerbate their perceptions of fear of hypoglycemia [23-27]. Our theoretical model of caregiver fear of hypoglycemia then proposes that greater caregiver fear of hypoglycemia relates to hypoglycemia avoidance behaviors, including maintaining blood glucose levels above the recommended range, treating blood glucose levels that are within the target range, and delaying or reducing insulin doses [16,28]. Our theoretical model of caregiver fear of hypoglycemia suggests that when caregivers engage in more hypoglycemia avoidance behaviors, these maladaptive coping strategies lead to chronically higher blood glucose levels, more glycemic variability, and increased risk for T1D-related complications for children [29,30]. Thus, our model suggests that caregiver fear of hypoglycemia may function as a barrier to optimal glycemic control and should be a target of behavioral interventions for families of young children with T1D.

In-person clinic visits have been a mainstay of behavioral interventions for families of youth with T1D, but this approach also presents logistical barriers (eg, travel, time, and cost). For this reason, the use of technology-based delivery methods has increased both in research and clinical settings. Access to mobile technology and the internet is ubiquitous in the United States. Current estimates suggest that 90% of American adults use the internet, 81% own smartphones, and 73% have high-speed internet access at home [31,32]. To date, most technology-based T1D interventions have focused on T1D management in adolescents and young adults versus families of young children, and the interventions have used email and text message support [33-35], websites and phone apps [36-44], and telephone counseling or video-based telehealth [45-51]. Yet these technology-based interventions suggest that it may be highly feasible and efficacious to use technology to intervene in families of youth with T1D. Moreover, using technology to intervene may be more scalable than in-person clinic delivery and enable behavioral interventions to reach a broader patient population, including families living in rural and underserved locations.

This study will fill an existing gap in the current T1D literature by developing and testing a video-based telehealth intervention to reduce caregiver fear of hypoglycemia in families of young children. Reducing Emotional Distress for Childhood Hypoglycemia in Parents (REDCHiP) uses a cognitive behavioral framework, T1D education, and behavioral parenting support to directly address caregiver fear of hypoglycemia and reduce their reliance on maladaptive coping strategies (including hypoglycemia avoidance behaviors) with the potential for downstream positive effects on young children’s T1D management and glycemic control. This paper outlines how we intend to examine the effectiveness of REDCHiP in reducing parenting stress and fear of hypoglycemia compared with an attention control intervention in a randomized clinical trial and obtain information about the intervention’s feasibility, acceptability, and impact on child glycemic control.

### Objectives

The aims of this randomized clinical trial are to (1) evaluate whether caregivers who receive the REDCHiP intervention report reductions in parenting stress and fear of hypoglycemia immediately posttreatment compared with caregivers who receive the attention control intervention; (2) evaluate whether children of caregivers who receive the REDCHiP intervention have lower HbA_1c_ and less glycemic variability posttreatment compared with children of caregivers who receive the attention control intervention; and (3) examine whether maintenance reductions in parenting stress and caregiver fear of hypoglycemia and child HbA_1c_ occur 3 months’ posttreatment for families receiving the REDCHiP intervention. Based on preliminary data, our primary hypotheses are (1) caregivers who receive REDCHiP will report reductions in parenting stress and fear of hypoglycemia compared with caregivers who receive the attention control intervention and (2) children of caregivers who receive REDCHiP will achieve more optimal glycemic control than children of caregivers who receive the attention control intervention.

## Methods

### Development of the Reducing Emotional Distress for Childhood Hypoglycemia in Parents Intervention

REDCHiP includes a cognitive behavioral framework based on the conceptualization that caregiver fear of hypoglycemia is a type of specific phobia. Individuals with specific phobias are “fearful or anxious about or avoidant of circumscribed objects or situations” and experience “fear, anxiety, or avoidance [that] is almost always immediately induced by the phobic situation, to a degree that it is persistent and out of proportion to the actual risk posed” [52]. Cognitive behavioral therapy plus systematic desensitization and exposures is a well-studied, evidence-based treatment for specific phobias in adults and demonstrates reductions in fear to a subclinical level in 90% of cases [53]. In REDCHiP, caregivers learn to recognize and alter thoughts and behaviors driven by fear of hypoglycemia and gain new behavioral parenting strategies and coping strategies to help them manage their fear. REDCHiP consists of 7 group and 3 individual sessions that are each 30 to 60 minutes in duration (Table 1).

We previously pilot-tested REDCHiP using a video-based telehealth approach. A total of 36 families completed the pilot intervention with low attrition (ie, 14%), high attendance (ie, 94%), and high caregiver-reported satisfaction [54]. Qualitatively, caregivers reported positive increases in knowledge, fear awareness, coping, and confidence and satisfaction with the support they received and the new behavioral parenting skills they learned from the REDCHiP intervention [54]. Quantitatively, caregivers experienced significant reductions in fear of hypoglycemia, parenting stress, and T1D-related distress [55]. Moreover, REDCHiP significantly reduced caregiver fear of hypoglycemia as compared against a waitlist control group, thus establishing preliminary efficacy for the REDCHiP intervention [55].

**Table 1:** Overview of the Reducing Emotional Distress for Childhood Hypoglycemia in Parents intervention.

Session No.	Format	Content
1-3, 5, 7, 9-10	Group	Hypoglycemia fear is a type of phobia and behavioral parent training, cognitive behavioral framework (eg, cognitive and behavioral responses to fear) and adaptations, diabetes education (eg, managing blood glucose levels and recognizing patterns in blood glucose)
4, 6, 8	Individual	Building a fear hierarchy and guided exposure, diabetes education (eg, recognizing your child’s symptoms of high and low glucose), challenging nighttime fear, problem-solving type 1 diabetes mellitus challenges

### Attention Control Development

We used two approaches to determine the content of our attention control intervention. First, parents of young children (ages 1 to 6 years) provided input by reviewing a list of potential topics and rating each topic on a 3-point scale according to its degree of relevance to them (not relevant, somewhat relevant, or very relevant). We then selected the topics identified as very relevant by the majority of caregivers. Second, we asked 5 experts in young child development and clinical psychology to review our initial list of topics and the list of topics caregivers provided and provide recommendations for additional developmentally and age-appropriate topics. The final list of sessions includes topics relevant to young children (eg, developmental milestones, child health and safety, starting school), positive parenting strategies, and early literacy; caregivers do not learn about T1D-related topics. To complement the REDCHiP format, the attention control intervention consists of 7 group and 3 individual sessions that are each 30 to 60 minutes in duration (Table 2).

**Table 2:** Overview of the attitude control intervention.

Session No.	Format	Content
1-3, 5, 7, 9-10	Group	Behavioral parent training, early childhood milestones and experiences, literacy, communication, learning through play
4, 6, 8	Individual	Early childhood safety topics (eg, injury prevention, car seat safety), creating a learning environment, establishing childhood routines and structure

### Design Considerations and Potential Challenges

We carefully designed the trial to reduce the impact of several potential challenges. First, we anticipated a challenge in recruiting an adequate sample because in very young children, T1D occurs at a prevalence of 0.29 per 1000 patients [56]. Therefore, we designed a multisite trial, which should enable us to adequately recruit our anticipated sample. Second, we anticipated barriers related to family availability and scheduling. To minimize this barrier, we designed the trial to deliver treatments via telehealth and include an option for families to complete study visits from home. Third, based on our pilot trial, we anticipated that a few eligible families (<10%) might not own a compatible device or have internet connectivity. Therefore, we have the flexibility in our trial design to loan web-enabled tablets to families. Fourth, we considered the possibility that some families might not find the intervention content or telehealth delivery favorable and will withdraw. To minimize negative trial effects due to attrition, we plan to recruit 180 families, which allows for a 20% attrition rate.

### Ethics and Dissemination

This is a multisite trial and follows National Institutes of Health guidelines to establish and operate within a single institutional review board (IRB) for final study monitoring. All research personnel will complete certification in responsible conduct of research, good clinical practice, and safe handling of biological samples. To minimize risk for participating families, we will inform all potential families of the purpose, procedures, and amount of time required to participate in the trial. We will minimize risk of breach of confidentiality by using a Health Insurance Portability and Accountability Act (HIPAA)-compliant telehealth platform and reviewing a group confidentiality agreement at the first session. We will minimize the risk of child pain or emotional discomfort when collecting blood samples by allowing families to use their own lancet and/or coordinating the sample collection to occur just after a clinic-based finger stick. Finally, we will protect caregivers by reviewing their responses on the study surveys within 24 hours of completion and contacting those who report concerning levels of depressive or anxiety symptoms to provide information on treatment resources. The trial was registered at ClinicalTrials.gov [NCT03914547].

The REDCHiP intervention builds on our previous research examining fear of hypoglycemia in caregivers of young children with T1D and fills a critical gap in behavioral intervention research in these understudied families. Moreover, the group-based telehealth approach is relatively novel for pediatric T1D interventions and may be easily scalable if the trial results confirm efficacy. We plan to disseminate the results of this trial to the pediatric diabetes community and broader medical community through national and international presentations at relevant scientific meetings and peer-reviewed manuscripts. We believe that there will be greater use of telehealth to deliver behavioral treatments for families of youth with T1D in the future because of the increasing adoption of telemedicine parity laws across the United States [57] and increased affinity for technology-enabled solutions to common needs in younger generations [58], including the need for convenient health care access [59]. We believe the format and general content of REDCHiP may be amenable to caregivers of older youth with T1D who also struggle with fear of hypoglycemia. Last, we expect that REDCHiP is in line with initiatives from the National Institute of Diabetes and Digestive and Kidney Diseases, which call for the development of more family-centered, efficacious, cost-effective, and easily scalable behavioral health interventions [60].

### Participants

For this randomized clinical trial, we will recruit 180 families of young children with T1D. Inclusion criteria are child aged from 2 to 5.99 years, T1D diagnosis ≥6 months, and use of an intensive insulin regimen (eg, insulin pump or multiple daily injections). Exclusion criteria are caregivers of children on a conventional insulin regimen, children who have an allergy or sensitivity to the adhesive and/or skin preparation used for a CGM, children with a comorbid chronic condition (eg, renal disease), and caregivers who do not speak English.

### Recruitment

We will recruit into the study at least one caregiver (mother, father, or guardian) who is primarily involved in the child’s daily T1D management. Each site will apply standardized recruitment procedures approved by a single IRB to achieve the target sample. We will use a combination of in-clinic and telephone recruitment. Families who express interest in participating via telephone will complete IRB-approved telephone informed consent procedures including an approved eConsent developed in the Research Electronic Data Capture (REDCap) system [61,62]. Families recruited in person may complete an eConsent or standard paper consent.

### Randomization

This study will use a 2-arm randomized attention control design with 90 families recruited to each of the REDCHiP and attention control intervention conditions. We will stratify caregivers based on their child’s sex, child age (2 to 3 years versus 4 to 5 years) and CGM use (CGM or no CGM) and randomize to condition using blocks of eight. Recruitment and randomization will occur simultaneously across all participating sites using site-specific randomization envelopes prepared by the study biostatistician. This strategy will allow us to populate groups using caregivers from any site, thereby improving our recruitment efficiency and minimizing possible clinic effects.

### Study Visit Procedures

**Figure 2:**
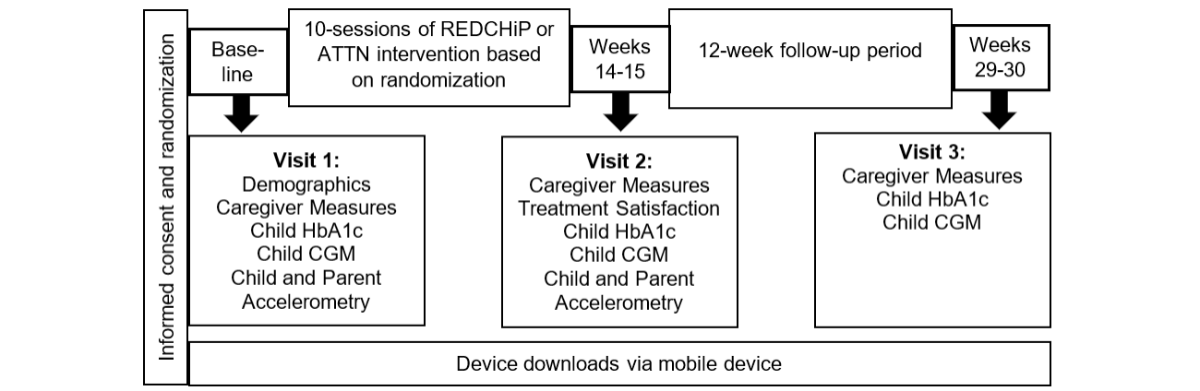
Participant timeline.

After informed consent and randomization, all families will complete study visit 1, during which caregivers will complete online surveys in REDCap and we will show caregivers (via a short video) how to place the research-grade accelerometer on their child’s nondominant wrist (to measure child physical activity) or ankle (to measure child sleep) and how to place the accelerometer on their own nondominant wrist to measure caregiver sleep. We will also teach caregivers how to upload glucometer and insulin pump data from home to a central study database using a commercially available data aggregating system (Glooko). In cases where caregivers cannot use the data aggregating system because of problems with device compatibility, we will collect .csv files. To measure children’s daily glucose levels, we will place a FreeStyle Libre Pro (Abbott Laboratories) CGM sensor on the child’s upper nondominant arm. We will collect a finger stick blood sample from children using a reliable mail-in kit to measure a baseline HbA_1c_ level. Finally, we will determine if caregivers need to borrow any equipment to participate in the intervention (eg, web camera, microphone, or tablet). After study visit 1, caregivers will begin participating in video-based telehealth sessions according to their group assignment (eg, REDCHiP or attention control intervention). Caregivers will participate in 10 video-based telehealth sessions administered during 13 weeks via a HIPAA-compliant telehealth platform that permits multiparticipant video teleconferencing so that each caregiver can both see and hear other caregivers. During weeks 14 to 15 of the trial, all families will engage in study visit 2 to complete posttreatment surveys online, collect accelerometry data from children and caregivers, collect child CGM data and finger stick blood sample to measure child HbA_1c_, and recover any loaned study-related devices. Approximately 12 weeks after study visit 2, all families will complete study visit 3, which will involve final data collection: online surveys, child CGM data, and a finger stick blood sample to measure child HbA_1c_ (Figure 2 and Table 3).

Of note, the study protocol will enable families to complete study visits either in their home, at the diabetes clinic, or at another location (eg, library). Additionally, our protocol includes strategies to retain families in the study in the rare event that they move away (eg, telehealth, online surveys, mail-in HbA_1c_ kits) or recruit families who self-refer based on word of mouth or ClinicalTrials.gov. All participating caregivers and children will receive compensation for study visits 1 through 3. As needed, the study will use prepaid postage boxes for families to return study-related devices (eg, accelerometers and CGM sensors) to further reduce the burden on participating families.

**Table 3:** Study outcome measures.

Outcome		Measure	Assessment schedule
**Child**			
	T1D^a^ treatment and history	Collect demographics; caregivers complete T1D History Questionnaire including caregiver report of family/child demographics, child insulin regimen, CGM^b^ use, and history of T1D complications	Visit 1
	Child physical activity	Calculate daily moderate to vigorous physical activity and sedentary time based on age-specific cutoffs using accelerometer data	Visits 1, 2
	Sleep	Calculate TST^c^, sleep latency, and sleep efficiency (TST/total time in bed); caregivers complete online sleep log to verify child sleep versus periods of wakeful inactivity	Visits 1, 2
	Glycemic control	Collect children’s blood samples using a finger stick capillary sampling kit (with or without mail-back box) developed at one of the study sites and record hemoglobin A_1c_; we will send samples to a central laboratory for processing using an automated G8 Analyzer^d^ with a reference range of 4.0% to 6.0%; this method has demonstrated reliability with a correlation of 0.98 relative to fresh venous samples	Visits 1, 2, 3
	Glucose variability	Calculate percentage above, below, and within target range (target: 70 to 180 mg/dL) and mean and standard deviation of daily glucose using FreeStyle Libre Pro^e^ data	Visits 1, 2, 3
	Child treatment engagement	Calculate frequency of self-monitoring blood glucose and mealtime (bolus) insulin use [63] using device data (eg, glucometer and insulin pump)	Visits 1, 2, 3
**Caregiver**			
	Hypoglycemia fear	Calculate total, worry, and behaviors scores from the HFS-PYC^f^ [15,26]	Visits 1, 2, 3
	Parenting stress	Calculate stress frequency and stress difficulty scores from the PIP^g^ [64]	Visits 1, 2, 3
	Depressive symptoms	Calculate total score from the CES-D-R^h^ [65]	Visits 1, 2, 3
	Anxiety	Calculate total score from PROMIS-A^i^ [66]	Visits 1, 2, 3
	Psychopathology	Calculate the depression, anxiety, and somatization scores and the Global Severity Index from the BSI-18^j^ [67]	Visits 1, 2, 3
	Sleep	Calculate TST, sleep latency, and sleep efficiency (TST/total time in bed) using accelerometer data; caregivers will also complete an online sleep log and the PROMIS-S^k^ [68], and we will calculate total score	Visits 1, 2 (Visits 1, 2, 3 for PROMIS-S)
	Treatment satisfaction	Calculate total score from the Treatment Satisfaction Questionnaire	Visit 2

^a^T1D: type 1 diabetes mellitus.

^b^CGM: continuous glucose monitor.

^c^TST: total sleep time.

^d^G8 analyzer: G8a High-Performance Liquid Chromatography Hemoglobin A_1c_ Analyzer (Tosoh Bioscience Inc).

^e^FreeStyle Libre Pro: FreeStyle Libre Pro Flash Glucose Monitoring System (Abbott Laboratories).

^f^HFS-PYC: Hypoglycemia Fear Survey–Parents of Young Children.

^g^PIP: Pediatric Inventory for Parents.

^h^CES-D-R: Center for Epidemiological Studies–Depression Scale Revised.

^i^PROMIS-A: Patient-Reported Outcomes Measurement Information System–Anxiety short form.

^j^BSI-18: Brief Symptom Inventory–18.

^k^PROMIS-S: PROMIS Sleep Disturbance and Sleep-Related Impairment.

### Data Analysis Plan

We will measure parenting stress and caregiver fear of hypoglycemia using the Pediatric Inventory for Parents [64] and the Hypoglycemia Fear Survey–Parents of Young Children [15,26], respectively. We will use child HbA_1c_ levels and percentage of time in range (eg, 70 to 180 mg/dL) to examine child glycemic control and glycemic variability, respectively. To test for treatment outcomes, we will model study visit 2 scores as a function of visit 1 scores, condition (REDCHiP versus attention control intervention), and selected covariates (eg, race/ethnicity, pump use) in mixed models that include a random intercept to account for clustering of participants within group cohorts. We will accommodate nonnormal outcome variables with log transformation, modeling with an appropriate generalized mixed model or nonparametric test. We will test the effect of condition based on whether the 95% confidence interval for the condition coefficient includes zero (equivalent to a 2-sided test at α=.05). To determine long-term treatment effects, we will test for sustained improvement on outcome variables by modeling scores at study visit 3 as a function of visit 1 scores and condition (REDCHiP versus attention control intervention). To control the overall rate of type I errors, we will conduct these analyses only for variables with a statistically significant condition effect in our primary analysis of treatment outcomes.

### Power Analysis

We anticipate recruiting 180 families, allowing for a 20% attrition rate and resulting in a final goal of completing the trial with at least 144 families. We assessed power using a simulation study; each simulated dataset contained 40 clusters (20 REDCHiP, 20 attention control intervention) of 4 to 5 participants each. We simulated posttreatment scores using the following model: Y_ij_ = rX_ij_ + u_i_ + ES×Condition_i_ + ε_ij_, where Y_ij_ is the posttreatment score for the *j*th participant in the *i*th cluster, r is the within-cluster correlation between baseline and posttreatment scores (set to .72 based on pilot data), X_ij_ is the *ij*th participant’s baseline score, u_i_ is the normally distributed random intercept for the *i*th cluster, ES is the standardized effect size (set to 0.6 standard deviations based on pilot data), Condition is an indicator (1 for REDCHiP, 0 for attention control intervention), and ε is a normally distributed error term. The variances of u_i_ and ε_ij_ were set to yield a within-condition intraclass correlation coefficient of .10. We fit a mixed model with a random cluster intercept and baseline score and condition as predictors to each of 1000 simulated data sets. To compute estimated power we took the average number of data sets for which the condition was statistically significant in a 2-sided test at α=.05. Based on the indicated parameter values and sample size, estimated power was 83%. For 40 groups averaging 3.6 families each, estimated power was 90%. Recognizing this trial will include a control group, which could attenuate our REDCHiP effect, if we conservatively reduce the standardized effect size to 0.4 standard deviations, estimated power is 85%.

## Results

Recruitment began in July 2019, and enrollment is ongoing. The first wave of the intervention began in December 2019. We anticipate completing enrollment in 2023. Final reporting of results will occur within 12 months of the primary completion date.

## Discussion

### Limitations

This trial will focus on families of young children with T1D and exclude children who are on conventional insulin therapy, which could limit some of its generalizability. However, we anticipate little negative impact from this decision because intensive insulin therapy is now the gold standard regimen for individuals with T1D. An examination of maintenance effects of REDCHiP up to 3 months’ posttreatment will occur, but we will not be able to draw inferences regarding longer term effects. Our trial includes numerous outcome measures, which increases the risk of missing data. We have attempted to reduce the risk of missing data through device use (eg, accelerometry to measure physical activity and sleep) and online questionnaire access and data sharing. We intend to give caregivers free and accessible technology support before and during telehealth visits in order to reduce any disruptions due to difficulties with the telehealth platform. We intend to stratify our samples according to child sex, age, and CGM status to control for any effect of these variables in our final models. Yet there may be other demographic variables we will not be able to control for a priori that moderate caregiver fear of hypoglycemia, and we may need to adjust for those in our final analyses. In addition, we intend to identify whether young children are using a hybrid closed loop or predictive low-glucose management system via caregiver report or electronic health record review and may adjust for these variables in our final analyses.

### Conclusions

Our experience studying fear of hypoglycemia in families of young children with T1D reveals that this is a common fear for caregivers that is related to poorer quality of life and suboptimal child glycemic control. Our initial pilot study revealed promising effects for using cognitive behavioral techniques and a group-based telehealth approach to reduce caregiver fear of hypoglycemia. If this larger, multisite trial confirms the efficacy of REDCHiP and the acceptability and feasibility of our telehealth delivery method, future directions will focus on widespread dissemination of the REDCHiP intervention and implementation across a variety of clinic settings.

## Abbreviations

CGM: continuous glucose monitor
HbA_1c_: glycated hemoglobin
HIPAA: Health Insurance Portability and Accountability Act
IRB: institutional review board
REDCap: Research Electronic Data Capture
REDCHiP: Reducing Emotional Distress for Childhood Hypoglycemia in Parents
T1D: type 1 diabetes mellitus
